# Comparative biochemical analysis of HIV-1 subtype B and C integrase enzymes

**DOI:** 10.1186/1742-4690-6-103

**Published:** 2009-11-11

**Authors:** Tamara Bar-Magen, Richard D Sloan, Verena H Faltenbacher, Daniel A Donahue, Björn D Kuhl, Maureen Oliveira, Hongtao Xu, Mark A Wainberg

**Affiliations:** 1 McGill University AIDS Centre, Lady Davis Institute-Jewish General Hospital, Montreal, Quebec, Canada; 2 Department of Microbiology and Immunology, McGill University, Montreal, Quebec H3A 2T5, Canada; 3 Division of Experimental Medicine, McGill University, Montreal, Quebec H3A 2T5, Canada

## Abstract

**Background:**

Integrase inhibitors are currently being incorporated into highly active antiretroviral therapy (HAART). Due to high HIV variability, integrase inhibitor efficacy must be evaluated against a range of integrase enzymes from different subtypes.

**Methods:**

This study compares the enzymatic activities of HIV-1 integrase from subtypes B and C as well as susceptibility to various integrase inhibitors *in vitro*. The catalytic activities of both enzymes were analyzed in regard to each of 3' processing and strand transfer activities both in the presence and absence of the integrase inhibitors raltegravir (RAL), elvitegravir (EVG), and MK-2048.

**Results:**

Our results show that integrase function is similar with enzymes of either subtype and that the various integrase strand transfer inhibitors (INSTIs) that were employed possessed similar inhibitory activity against both enzymes.

**Conclusion:**

This suggests that the use of integrase inhibitors against HIV-1 subtype C will result in comparable outcomes to those obtained against subtype B infections.

## Background

Integration of viral cDNA into the host genome is one of the definitive features of retroviral replication. Integration is mediated by the HIV *pol-*encoded integrase enzyme. Recently, integrase inhibitors have been added to the arsenal of antiviral drugs used in therapy. RAL (Merck) was the first integrase inhibitor to be approved by the US Food and Drug Administration (FDA) after clinical trials demonstrated that this drug promoted a rapid and sustained antiretroviral effect [1]. EVG (GS-9137, Gilead), another integrase inhibitor, is currently in phase III clinical trials [2]. Other integrase inhibitors, such as MK-2048 (Merck), are still in pre-clinical development.

Integrase inhibitors are active against both B- and non-B subtypes in therapy [3,4]. Subtype C variants are responsible for approximately 50% of global infections, mostly in Sub-Saharan Africa and India [5]. It is therefore important to determine whether the integrase enzymes of different HIV-1 subtypes behave in a parallel manner to one another and whether they respond similarly to the use of integrase inhibitors of HIV-1 replication.

After viral entry and reverse transcription, reverse-transcribed double-stranded blunt-ended DNA is incorporated into the host cell genome through two catalytic activities mediated by integrase: 3' end processing and strand transfer [6,7]. During 3' end processing, a dinucleotide adjacent to the conserved 3' terminal CA is excised from the 3' end of the recently reverse transcribed HIV-1 DNA genome, generating 3' hydroxyl ends. During the strand transfer reaction, both newly generated 3' ends are covalently linked to target DNA in a concerted fashion via a one-step transesterification reaction [8]. *In vitro*, integrase can also catalyze two additional reactions: disintegration and specific internal endonucleolytic cleavage [9,10].

Variability between different HIV-1 integrases at an amino acid level is low, ≈ 8-12%. However, sites of amino acid differences between subtypes are often close to resistance-related amino acids. We were therefore interested in analyzing whether such minor differences might be important in differential acquisition of INSTI resistance mutations in a subtype-specific manner [11]. Furthermore, natural polymorphisms in non-B integrase proteins might alter INSTI binding or activity [12,13]. An *in silico *comparison of subtype B and CRF A/G integrase predicted that polymorphisms within subtypes might affect structure and substrate binding characteristics of IN enzymes [13]. In this study, we compared the enzymatic activities of subtype B and C recombinant integrases in the context of inhibition by RAL, EVG, and the novel INSTI MK-2048.

## Results

### Purification of active subtype C integrase

**Figure 1:**
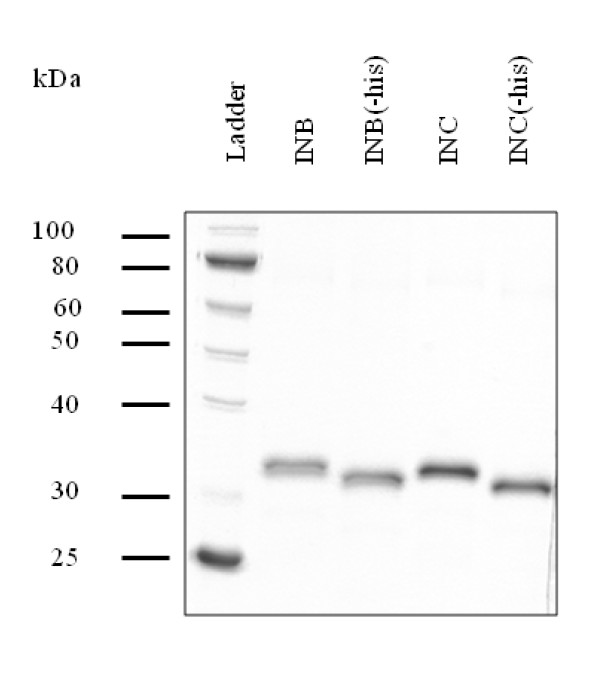
**Purification of recombinant subtype B and C integrase enzymes**. N-terminal His tags of the enzymes were removed from purified subtype B and C recombinant proteins by thrombin cleavage. Lane 1, protein ladder (10-250 kDa) (New England Biolabs); INB, subtype B integrase; INC, subtype C integrase.

Subtype C integrase was PCR amplified from the pINDIE-C1 molecular clone and introduced into the expression vector pET-15B, replacing the ORF of subtype B integrase previously cloned by Bushman *et al*. [14]. To increase the solubility of subtype C recombinant proteins, two amino acid changes were introduced: a phenylalanine at codon 185 was changed to a histidine, and a cysteine at codon 280 was changed to a serine. These changes mimic those previously introduced into subtype B integrase to increase solubility and are known to not affect catalytic activity [15,16]. Expression and purification of the subtype B and C integrase enzymes were performed simultaneously as previously described for subtype B integrase [15] with minor modifications. Subtype B and C integrases were successfully purified to > 95% homogeneity (Figure 1). The N-terminal His tag was removed from recombinant integrase enzymes by thrombin cleavage (Figure 1). When the enzymatic activities of both subtype B and C purified recombinant proteins in the presence or absence of the N-terminal His tag were compared, no difference was detected (data not shown). Therefore, all further experiments were orchestrated using recombinant integrase that did not undergo His tag removal.

### Biochemical properties of subtype C integrase

**Figure 2:**
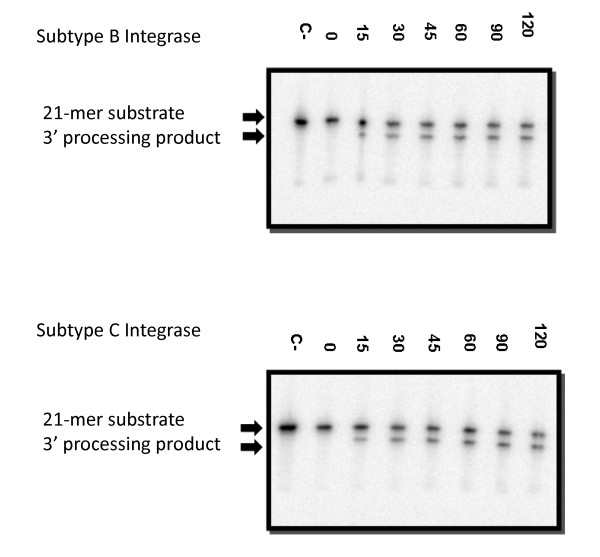
**3' Processing assay**. One representative reaction (out of five reactions) is illustrated. Recombinant enzyme was incubated at 37°C with templates (radiolabeled double stranded oligonucleotide INT1/2) for the indicated times up to 120 minutes. The 21-mer substrate and 19-mer 3' processing products are indicated.

**Figure 3:**
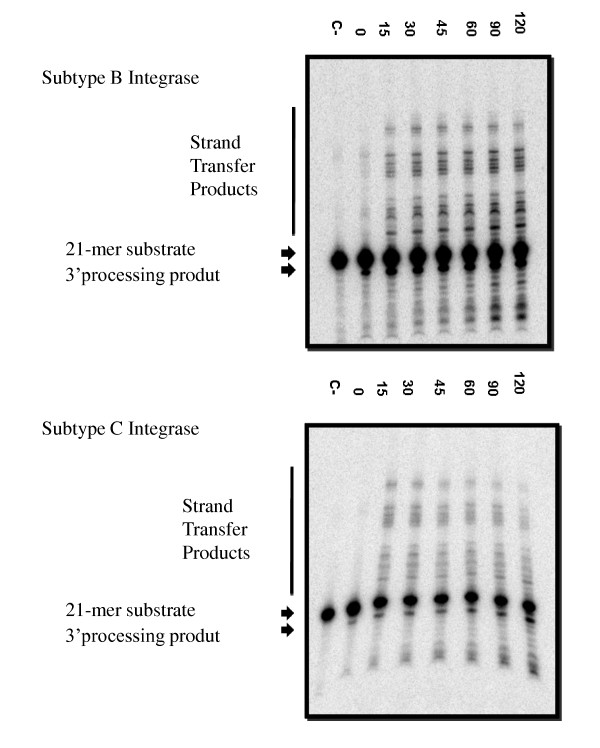
**Strand transfer assay**. One representative reaction (out of five reactions) is depicted. Recombinant integrase enzyme was incubated at 37°C for 3 minutes for the initial 3' processing reaction. T35/SK70, double stranded oligonucleotide substrate, was added and reaction tubes were incubated at 37°C for the indicated times up to 120 minutes. The 21-mer substrate, 19-mer 3' processing, and strand transfer products are indicated.

**Figure 4:**
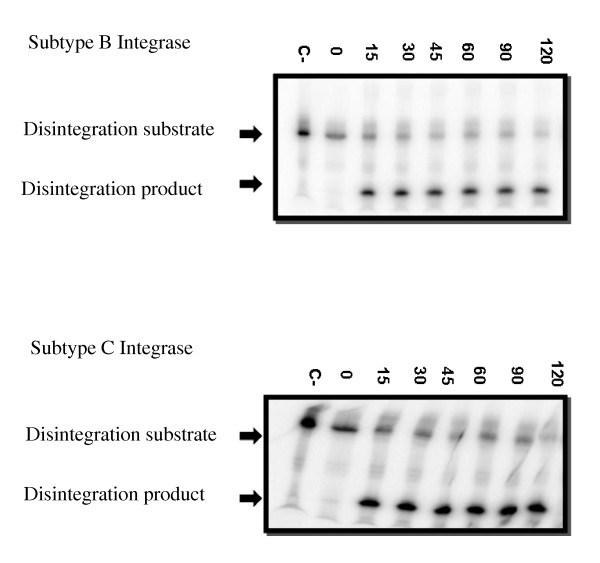
**Disintegration assay**. One representative reaction (out of five reactions) is portrayed. Recombinant enzyme was incubated at 37°C for the indicated times up to 120 minutes with disintegration template (radiolabeled oligonucleotide D). Disintegration template and product are indicated. (C-), Negative control lane without integrase enzyme. **Top panel**, subtype B integrase; **bottom panel**, subtype C integrase.

Integrase mediates the insertion of viral cDNA into host chromatin through two unique enzymatic activities: 3' processing and strand transfer [6,17]. Oligonucleotides that mimic the viral LTR ends can be utilized to analyze these two catalytic activities *in vitro*. First, subtype B and C integrases were tested for their ability to perform 3' processing (Figure 2) and strand transfer (Figure 3). Time course experiments show similar results for both enzymes. Disintegration was also analyzed and subtype C recombinant protein catalyzed this activity to a similar extent as did subtype B recombinant protein (Figure 4). These experiments confirm the activity of our subtype C purified recombinant protein.

### Subtype B and C enzymes are inhibited to a similar extent by RAL, MK-2048 and EVG

RAL and EVG are INSTIs with high specific activity against strand transfer [18,19]. MK-2048 is a prototype second-generation INSTI with a resistance profile that is distinct from RAL and EVG [20,21]. These three drugs have been reported to be approximately 100-fold less specific for the inhibition of 3' processing activity compared to strand transfer [18,22,23].

**Figure 5:**
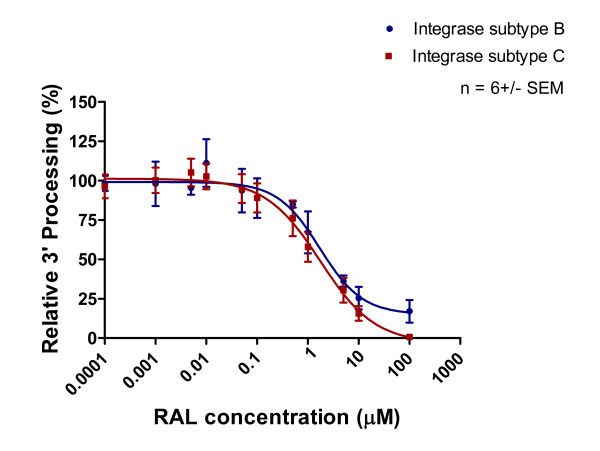
**Inhibition of 3' processing as a function of increasing RAL concentration**. Subtype B and C 3' processing activity (presented as relative percentage) in relation to increasing RAL concentration. This graph was prepared with GraphPad Prism 4.0, the combined result of quantification and analyses of at least 3 independent experiments.

**Figure 6:**
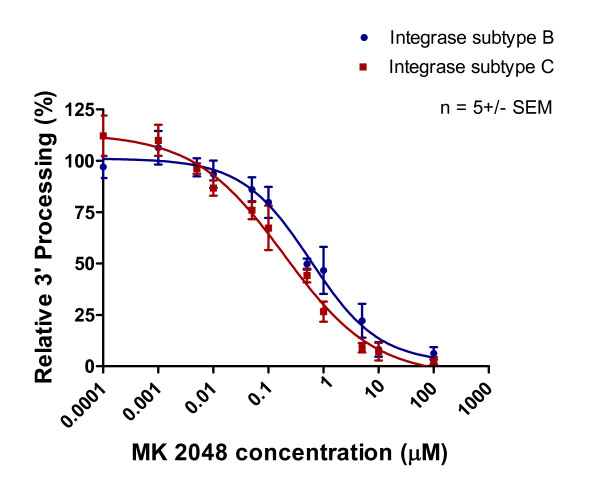
**Inhibition of 3' processing as a function of increasing MK-2048 concentration**. Subtype B and C 3' processing activity (presented as relative percentage) in relation to increasing MK-2048 concentration. This graph was prepared with GraphPad Prism 4.0, the combined result of quantification and analyses of at least 3 independent experiments.

**Figure 7:**
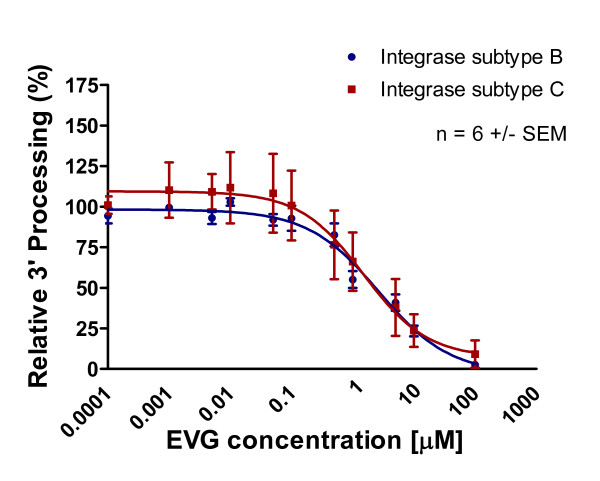
**Inhibition of 3' processing as a function of increasing EVG concentration**. Subtype B and C 3' processing activity (presented as relative percentage) in relation to increasing EVG concentration. This graph was prepared with GraphPad Prism 4.0, the combined result of quantification and analyses of at least 3 independent experiments.

Purified recombinant subtype B and C integrase enzymes were incubated with increasing concentrations of integrase inhibitors and corresponding templates. The results of Table 1 and Figures 5, 6 and 7 show that 3' processing mediated by recombinant enzymes of both subtypes was inhibited to a similar extent **(p > 0.05) **by all three drugs in the presence of MnCl_2_. The inhibition of 3' processing required much higher concentrations of integrase inhibitors than those needed to block strand transfer for both subtype enzymes (Table 1), consistent with previously reported data for subtype B integrase [18].

**Table 1:** IC_50 _values for RAL, EVG and MK-2048 for subtype B and subtype C integrase in Mn^2+^-based enzymatic assays.

	**3' Processing IC_50_^a^**		**Strand Transfer IC_50_^a^**	
	
	**Subtype B**	**Subtype C**	**Subtype B**	**Subtype C**
**RAL(μM)**	1.71(0.7-4.5)	1.75(0.7-3.9)	0.37(0.2-0.8)	0.15(0.09-0.3)
**MK-2048(μM)**	0.58(0.28-1.20)	0.19(0.09-0.39)	0.075(0.04-0.14)	0.08(0.03-0.2)
**EVG(μM)**	2.66(1.44-4.91)	1.5(0.29-7.74)	0.014(0.003-0.07)	0.018(0.006-0.05)

^a^All differences between subtypes were not statistically significant. 95% Confidence Intervals are indicated.

**Figure 8:**
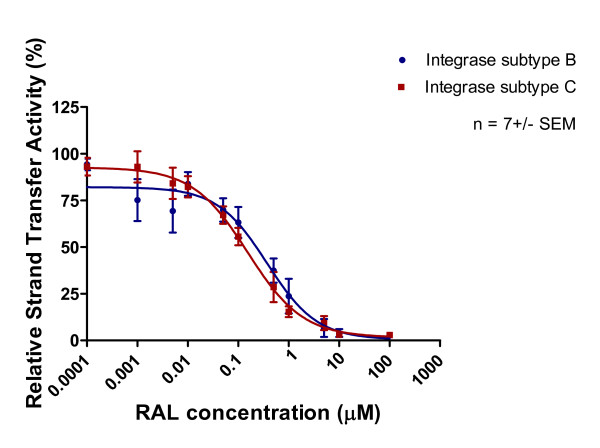
**Inhibition of strand transfer as a function of increasing RAL concentration**. Subtype B and C strand transfer activity (presented as relative percentage) in relation to increasing RAL concentration. This graph was prepared with GraphPad Prism 4.0, the combined result of quantification and analyses of at least 3 independent experiments.

**Figure 9:**
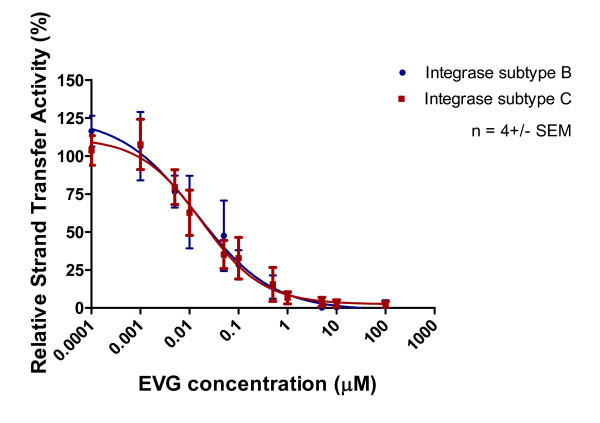
**Inhibition of strand transfer as a function of increasing EVG concentration**. Subtype B and C strand transfer activity (presented as relative percentage) in relation to increasing EVG concentration. This graph was prepared with GraphPad Prism 4.0, the combined result of quantification and analyses of at least 3 independent experiments.

**Figure 10:**
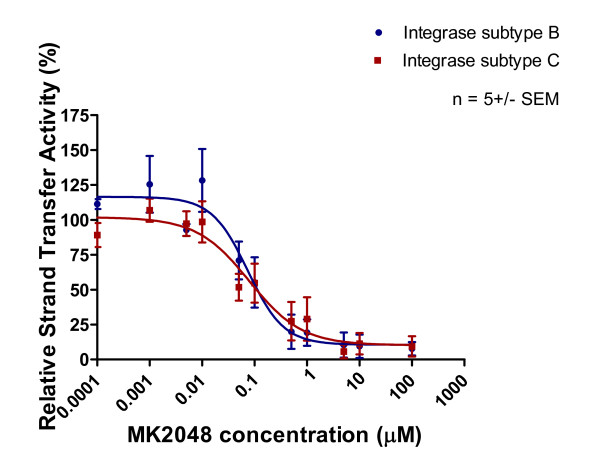
**Inhibition of strand transfer as a function of increasing MK-2048 concentration**. Subtype B and C strand transfer activity (presented as relative percentage) in relation to increasing MK-2048 concentration. This graph was prepared with GraphPad Prism 4.0, the combined result of quantification and analyses of at least 3 independent experiments.

The strand transfer activity of subtype B and C recombinant proteins was inhibited by all three inhibitors. The IC_50 _values of RAL for subtype B and C integrase strand transfer were 0.37 μM and 0.15 μM, respectively, in assays that employed Mn^2+ ^as the cation (Figure 8, Table 1). The IC_50 _values for EVG inhibition of strand transfer in Mn^2+^-based assays were 0.014 μM and 0.018 μM for the subtype B and C enzymes, respectively (Figure 9, Table 1). The IC_50 _values for MK-2048 against subtype B and C enzymes were 0.075 μM and 0.08 μM, respectively (Figure 10, Table 1).

**Figure 11:**
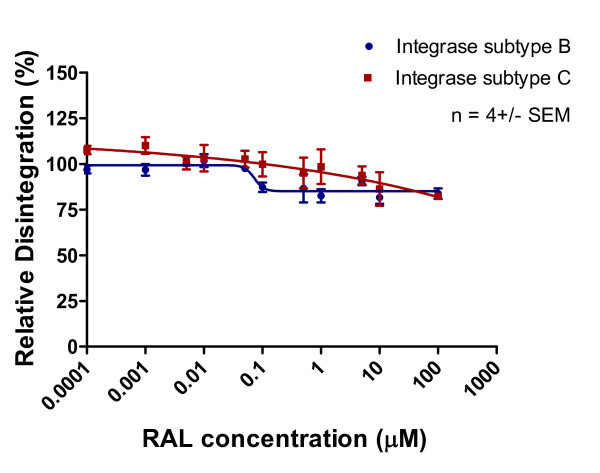
**Inhibition of disintegration as a function of increasing RAL concentration**. Subtype B and C disintegration activity (presented as relative percentage) in reaction to increasing RAL concentration. This graph was prepared with GraphPad Prism 4.0, the combined result of quantification and analyses of 3 independent experiments.

**Figure 12:**
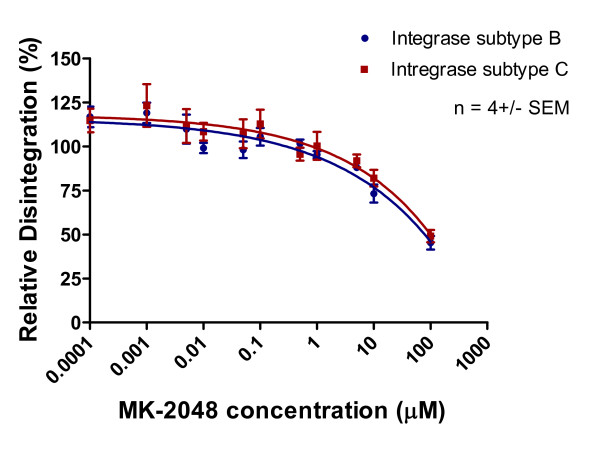
**Inhibition of disintegration as a function of increasing MK-2048 concentration**. Subtype B and C disintegration activity (presented as relative percentage) in reaction to increasing MK-2048 concentration. This graph was prepared with GraphPad Prism 4.0, the combined result of quantification and analyses of 3 independent experiments.

**Figure 13:**
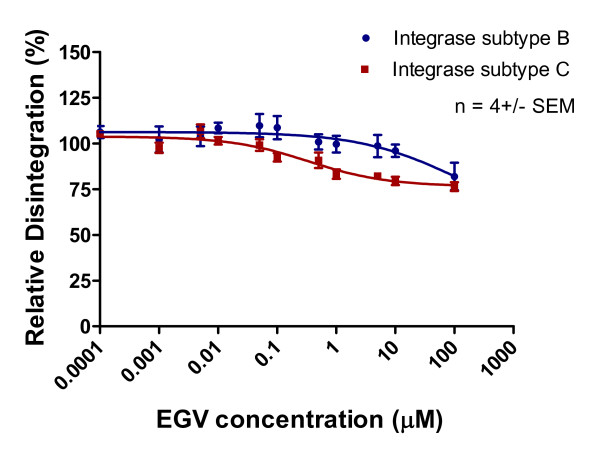
**Inhibition of disintegration as a function of increasing EVG concentration**. Subtype B and C disintegration activity (presented as relative percentage) in reaction to increasing EVG concentration. This graph was prepared with GraphPad Prism 4.0, the combined result of quantification and analyses of 3 independent experiments.

Disintegration was inhibited by high concentrations of MK-2048 to a comparable extent with both subtype B and C enzymes (Figures 11, 12, 13). In contrast, neither RAL nor EVG had much effect on this process, which is a discovery that is consistent with work by others [22]. We also evaluated strand transfer in the presence of MgCl_2 _rather than MnCl_2 _and obtained similar IC_50 _values (p > 0.05) for each of subtype B versus C enzymes with each of RAL, EVG and MK-2048 in a microtiter plate system [24] (Table 2). Consistent with previous observations, IC_50 _values were lower when these reactions were performed with MgCl_2 _than with MnCl_2 _[2,24].

**Table 2:** IC_50 _values for RAL, EVG and MK-2048 for subtype B and subtype C integrase in Mg^2+^-based enzymatic assays.

	**Strand Transfer IC_50_**	
	
	**Subtype B^a^**	**Subtype C^a^**
**RAL (μM)**	0.0047 (0.0013-0.0064)	0.0037 (0.0011-0.0086)
**MK-2048 (μM)**	0.0047 (0.0021-0.010)	0.0023 (0.001-0.007)
**EVG (μM)**	0.0017 (0.0009-0.0051)	0.0011 (0.0002-0.021)

^a^All differences between subtypes were not statistically significant. 95% Confidence Intervals are indicated.

Inhibition of replication by integrase inhibitors was also evaluated in cell culture based assays using cord blood mononuclear cells (Table 3). Subtype B and C clinical isolates were inhibited to a similar extent by each of RAL, EVG and MK-2048.

**Table 3:** EC_50 _values for RAL, MK-2048 and EVG for subtype B and C viruses cultured in cord blood mononuclear cells.

Subtype B	EC_50 _Values (μM)		
**Viruses**	**RAL (μM)**	**MK-2048 (μM)**	**EVG (μM)**

5326	0.0243 ± 0.0025	0.0072 ± 0.0021	ND^a^
5331	0.0056 ± 0.0022	0.0010 ± 0.0002	0.0001 ± 0.00007
BK 132	0.0301 ± 0.0054	0.0148 ± 0.0032	ND^a^
pNL4-3	0.0082 ± 0.0013	0.0027 ± 0.0009	0.0009 ± 0.0003
IIIb	0.0012 ± 0.0005	0.0003 ± 0.0001	0.0018 ± 0.0006
**Subtype C**	**EC_50 _Values (μM)**		

**Viruses**	**RAL (μM)**	**MK-2048 (μM)**	**EVG (μM)**

Mole 03	0.0046 ± 0.0002	0.0011 ± 0.0001	ND^a^
96USNG31	0.0087 ± 0.0007	0.0015 ± 0.0001	0.0008 ± 0.0006
4742	0.0206 ± 0.0090	0.0033 ± 0.0020	0.0022 ± 0.0002
BG-05	0.0067 ± 0.0009	0.0022 ± 0.0007	0.0008 ± 0.00004
HB-1	0.0015 ± 0.0004	0.0007 ± 0.0005	0.0001 ± 0.00001

^a^ND = not done

## Discussion

Most HIV-1 patients are infected with non-B subtypes, most commonly subtype C [5], and subtype-specific differences in the development of drug resistance have been reported [25]. Therefore, it is important to understand the activity of enzymes of different subtypes. In our study, subtype B and C integrase enzymes were evaluated; and the activity of integrase inhibitors against them were compared, since a role for polymorphisms and structure-function differences between subtypes in drug resistance has been demonstrated [11,12,23].

Strand transfer inhibitors have been shown as efficient inhibitors of integration amongst a wide range of retroviruses [26]. *In silico *observations suggest that subtype-specific differences in regard to key amino acids in integrase, including those close to the catalytic site, may pose an effect on the binding of RAL [13,27,28]. Therefore, subtype-specific variations in DNA-binding domains could also affect the affinity of RAL for integrase. *In vitro*, subtype B and C recombinant proteins retain similar enzymatic capacities in the absence of drug (Figures 2, 3, 4), with comparable strand transfer, 3' processing and disintegration activities, as measured by time course experiments. We also show that RAL and EVG had similar effects against both subtype B and C integrase enzymes, regardless of whether Mg^2+ ^or Mn^2+ ^was used as a cation (Tables 1 and 2). In addition to the foregoing, we have evaluated the IC_50 _values of RAL, EVG and MK-2048 in cell-based assays using clinical isolates of viruses of either subtype B or subtype C origin (Table 3). No significant differences were observed between subtypes in regard to drug susceptibility. These findings are consistent with recent results on similarities vis-à-vis biochemical activity and susceptibility to antiretroviral drugs of reverse transcriptase enzymes derived from HIV-1 subtypes B and C [29].

## Conclusion

Our results provide biochemical and tissue culture evidence that integrase enzymes from HIV-1 subtypes B and C are inhibited by each of RAL, EVG and MK-2048 to a similar extent. These findings are supportive of the use of these inhibitors in patients infected with subtype C virus.

## Materials and methods

### Oligonucleotides and reagents

Oligonucleotides were purchased from Invitrogen and then PAGE purified. A 21-mer duplex was formed by annealing INT1 (5'-TGTGGAAAATCTCTAGCAGT-3') and INT2 (5'-ACTGCTAGAGATTTTCCACA-3'). The 35-mer duplex was produced by annealing T35 (5'-ACTATACCAGACAATAATTGTCTGGCCTGTACCGT-3') and SK70 (5'-ACGGTACAGGCCAGACAATTATTGTCTGGTATAGT-3'). The disintegration primer (5'-TGCTAGTTCTAGCAGGCCCTTGGGCCGGCGGCGCTTGCGCC-3') was heated to 95°C and slowly cooled to achieve its secondary structure [30].

RAL and MK-2048 were obtained from Merck Pharmaceuticals, Inc. EVG was obtained from Gilead Biosciences.

### Cloning and Mutagenesis

Subtype C integrase was PCR amplified from the molecular clone pINDIE-C1 (accession number: AB023804) and subcloned into the bacterial expression vector pET15B, replacing the subtype B integrase ORF kindly obtained from Dr. Robert Craigie, NIH.

The QuickChange II (Stratagene) site-directed mutagenesis kit was utilized for the introduction of the solubility mutations F185H and C280S into subtype C integrase. The primers utilized for this mutagenesis were INC-FF185H (5'-GCAGTATTCATTCACAATCATAAAAGAAAAGGGGGG-3'), INC-RF185H (5'-CCCCCCTTTTCTTTTATGATTGTGAATGAATACTGC-3'), INC-FC280S (5'-GCAGGTGCTGATTCTGTGGCAGGTAGACAG-3') and INC-RC280S (5'-CTGTCTACCTGCCACAGAATCAGCACCTGC-3').

### Protein purification

Wild type and mutant His-tagged integrase proteins were expressed in *Escherichia coli *BL21(DE3) and purified under non-denaturing conditions. Bacterial cultures were grown at 37°C. When cultures of BL21 achieved an optical density of 0.5 at 600 nm, protein expression was induced by the addition of isopropyl-β-D-thiogalactopyranoside (IPTG) to a final concentration of 1 mM. The cultures were incubated for 3 hours at 37°C, centrifuged (5000 rpm for 12 min), and frozen at -80°C. The pellets were resuspended in lysis buffer (20 mM Hepes pH 7.5, 100 mM NaCl, 2 mM β-ME, protease inhibitors) and lysed by sonication. The lysates were centrifuged (12 500 rpm for 30 min), the supernatants discarded, and the pellets resuspended in binding buffer (1 M NaCl, 20 mM imidazole, 20 mM Hepes pH 7.5, 2 mM β-ME, 100 μM ZnCl_2_, protease inhibitors). Following centrifugation at 12500 rpm for 30 min, the supernatants were incubated with nickel-nitrioltriacetic acid (Ni-NTA) agarose beads (Qiagen) for 1 hour at 4°C with mild agitation. Proteins were purified utilizing propylene columns (Qiagen). His-tagged integrase protein was then eluted, utilizing a gradient of increasing imidazole concentration (0-2 M) in elution buffer (1 M NaCl, 20 mM Hepes pH7.5, 10% glycerol, 2 mM β-ME, 100 μM ZnCl_2_). The eluates were analyzed on 10% SDS-polyacrylamide gels with Coomassie staining (Sigma-Aldrich). Proteins were dialyzed overnight at 4°C against 1 M NaCl, 200 mM Hepes pH 7.5, 100 μM ZnCl_2_, 10% glycerol and 2 mM DTT in dialysis cassettes (10,000 MWCO, ThermoScientific). The samples were aliquoted and fast frozen at -80°C. Protein concentration was measured by Bradford assay utilizing the Bradford Protein Assay kit (Bio-Rad Laboratories).

### Thrombin His tag exclusion

The His tags were removed from purified recombinant subtype B and C integrases using the Thrombin CleanCleave kit (Sigma) according to manufacturers' instructions. Proteins were dialyzed overnight at 4°C against 1 M NaCl, 200 mM Hepes pH 7.5, 100 μM ZnCl_2_, 10% glycerol and 2 mM DTT in dialysis cassettes (10,000 MWCO, ThermoScientific).

### 3' processing, strand transfer and disintegration assays evaluated in urea gels

Oligonucleotide-based assays were performed to measure integrase enzymatic activities. All oligonucleotide probes were gel purified and phenol-chloroform extracted. INT1 oligonucleotide was radiolabeled using the T4 Polynucleotide Kinase kit (Ambion) with [γ-^32^P] ATP (Perkin Elmer). Unincorporated nucleotides were discarded utilizing 'NucAway columns' (Ambion). A double stranded oligonucleotide substrate was obtained by mixing equal concentrations of INT1 and INT2, heating to 95°C and stepwise cooling to 37°C in 100 mM NaCl. The oligonucleotides T35 and SK70 were annealed to form a second double stranded oligonucleotide. INT1/2 mimicked the HIV-1 U5 long terminal repeat (LTR) and acted as a substrate for 3' processing. T35/SK70 acted as a site for integration of the processed INT1/2 fragment and mimicked host DNA.

Concentrations of enzymes used in the following reactions were optimized in a series of preliminary experiments. Integrase reactions were performed in a buffer containing 20 mM Hepes (pH 7.5), 30 mM NaCl, 1 mM dithiothreitol, 125 μM ZnCl_2 _and 0.125 pmol dsDNA substrate (INT1/2) in a final volume of 10 μl. Recombinant integrase (3.1 μM), and varying concentrations of integrase inhibitors or water/DMSO as control, were mixed and preincubated at 37°C for 15 min. 3' processing reactions were initiated by addition of 7.5 mM of MnCl_2 _and incubated at 37°C for 3 min unless otherwise indicated. For strand transfer reactions, 1.25 pmol of dsDNA template (T35/SK70) were added and the reaction mixture was further incubated at 37°C for 1 hour unless otherwise indicated. Reactions were stopped by adding 5 times the volume of gel loading dye (formamide containing 1% SDS, 0.25% bromophenol blue, and 0.25% xylene cyanol) and heating to 95°C. Reaction products were separated on 6% acrylamide, 7 M urea sequencing gels. Gels were dried and exposed utilizing phosphorimager screens (GE Healthcare) and scanned in a Molecular Dynamics Typhoon Phosphorimager (GE Healthcare). Product analysis and quantification were conducted using ImageQuant and GraphPad Prism 4.0 software. Quantification of standard error of the mean (SEM) was performed with GraphPad Prism 4.0 software.

### The use of microtiter plates for strand transfer analysis

A microtiter plate assay was utilized to evaluate MgCl_2_-mediated strand transfer as previously described [24,31]. Briefly, biotinylated oligonucleotides mimicking LTR donor DNA (5'-biotin-ACCCTTTTAGTCAGTGTGGAAAATCTCTAGCAGT and 5'-ACTGCTAGAGATTTTCCACACTGACTAAAAG) were immobilized onto black-colour Reacti-Bind Streptavidin-coated plates (ThermoFisher). 313 nM recombinant enzyme was bound to donor DNA on the plates in the presence of 25 mM MnCl_2 _and the plates were then washed to remove excess unbound enzyme. 3'-FITC labelled dsDNA (5'-TGACCAAGGGCTAATTCACT-FITC-3' and 3'-FITC-ACTGGTTCCCGATTAAGTGA-5'), used as a reaction target, was added to the wells in 25 mM Hepes (pH 7.8), 25 mM NaCl, 2.5 mM MgCl_2_, and 50 μg/mL BSA [24,32] and the plate was incubated at 37°C for 1 hour. Covalently linked target DNA was detected through use of an anti-FITC antibody conjugated to alkaline phosphatase (Roche) and a chemiluminescence substrate (CSPD Sapphire II, Applied Biosystems). Integrase inhibitors were added at increasing concentrations shortly before the addition of target DNA. Strand transfer was evaluated by chemiluminescence.

### Statistical Analysis

Unpaired two-tailed t-tests were used to examine statistical significance in subtype B versus subtype C integrase enzymatic assays using GraphPad Prism 4.0 software.

### Determination of activity of integrase inhibitors in cell culture

Recombinant viruses (subtype B) (pNL4-3 and IIIb) and viruses obtained from either our primary HIV infection cohort or from long-term infected patients (subtype B and C) were amplified as previously described [33,34]. Drug susceptibility was measured in cell culture-based phenotypic assays using cord blood mononuclear cells to determine the extent of in vivo HIV replication blockage by integrase inhibitors. 50% drug effective concentrations (EC50s) were determined for each of RAL, EVG and MK-2048 by monitoring the production of p24 antigen, as previously elaborated [33].

## Competing interests

The authors declare that they have no competing interests.

## Authors' contributions

TB designed all of the biochemistry and enzyme experiments performed in this assay. She wrote the first draft of the manuscript. RDS, DAD and BDK contributed to data management and interpretation. These individuals also contributed to the writing of the manuscript. VHF performed some of the biochemical analyses. HX was involved in preparation and purification of integrase enzymes. MO was responsible for tissue culture analyses. MAW supervised the project, secured funding toward its implementation and contributed to the writing of the manuscript.
